# Review of human-animal interactions and their impact on animal productivity and welfare

**DOI:** 10.1186/2049-1891-4-25

**Published:** 2013-07-15

**Authors:** Idrus Zulkifli

**Affiliations:** 1 Institute of Tropical Agriculture, and Department of Animal Science, Universiti Putra Malaysia, 43400, UPM, Serdang, Selangor, Malaysia

**Keywords:** Animal welfare, Fear, Human-animal interactions, Productivity, Stress

## Abstract

Humans and animals are in regular and at times close contact in modern intensive farming systems. The quality of human-animal interactions can have a profound impact on the productivity and welfare of farm animals. Interactions by humans may be neutral, positive or negative in nature. Regular pleasant contact with humans may result in desirable alterations in the physiology, behaviour, health and productivity of farm animals. On the contrary, animals that were subjected to aversive human contact were highly fearful of humans and their growth and reproductive performance could be compromised. Farm animals are particularly sensitive to human stimulation that occurs early in life, while many systems of the animals are still developing. This may have long-lasting impact and could possibly modify their genetic potential. The question as to how human contact can have a positive impact on responses to stressors, and productivity is not well understood. Recent work in our laboratory suggested that pleasant human contact may alter ability to tolerate various stressors through enhanced heat shock protein (hsp) 70 expression. The induction of hsp is often associated with increased tolerance to environmental stressors and disease resistance in animals. The attitude and consequent behaviour of stockpeople affect the animals’ fear of human which eventually influence animals’ productivity and welfare. Other than attitude and behaviour, technical skills, knowledge, job motivation, commitment and job satisfaction are prerequisites for high job performance.

## Introduction

Farm animals have undergone the process of domestication, a continuing genetic process aimed at modifying the animal’s behaviour, anatomy and physiology to suit mankind’s specific needs [1]. Hence, domestic animals should be adapted to man and captive environment. However, many farm animals still perceive contact with humans as an alarming predatory encounter and sudden changes in their physical and social environment as a frightening experience [2,3]. In modern production systems, there are regular periods of contacts between humans and animals such as during feeding and cleaning. Animals may respond to tactile, visual, olfactory, gustatory and auditory stimuli from humans. Even with considerable automation in intensive farming, animals are still subjected to some degree of human contact. The behaviour of stockpersons is an important factor in determining the degree of animals’ fear in humans and consequently the quality of the human-animal interaction [4]. Fear for humans is a major source of stress and may result is poor productivity in farm animals. This subject has been extensively reviewed previously [4-7].

The quality of human-animal interaction can have a profound impact on many facets of an animal’s physiology and behaviour. Interactions by humans may be neutral, positive or negative in nature. Regular positive contact with humans is desirable in both mammalian and avian species [8]. On the other hand, farm animals that were handled aversively were highly fearful of humans, distressed and consequently their welfare and productivity will be compromised [5]. There have been considerable reports of attempts to alter the physiology, behaviour and performance pigs, poultry and cattle through regular positive contact with humans at both laboratory and farm levels [6,9]. The aim of this paper is to review some of the research that shows impact of human-animal interactions on the productivity and welfare of farm animals.

### The concept of human-animal interactions

According to Estep and Hetts [10], human-animal interactions can be defined as the degree of relatedness or distance between animal and humans. A relationship develops between the stockperson and an animal in his / her care. The relationship requires mutual individual recognition. Animals may respond to tactile, visual, olfactory, gustatory and auditory stimuli from humans. The quality of human-animal interactions will determine whether the influence on an animal’s physiology and behaviour is desirable or otherwise [5]. There is the question of whether animals can discriminate one human to another human. Literature regarding the ability of farm animals to recognise individual people is inconsistent. A number of experiments suggested that farm animals respond the same way to different people. Hemsworth [11] compared the response of pigs to two different stockpersons which differed markedly in their nature of contact with pigs. The authors concluded that pigs were unable to differentiate between different people and aversive handling by one person made the animals fearful of all people. Jones [12] indicated that chicks that had been habituated to one person by a regimen of regular handling also showed less fear of similarly dressed but otherwise dissimilar people. On the contrary, other studies showed that pigs [13,14], laying hens [15] and sheep [16] were able to recognise individual people. Rybarczyk [17] suggested that dairy cows may recognise people by their faces [17].

### Modifying the stress and fear reactions in animals through human-animal interactions

Stress can be defined as any disruption of an animal’s homeostatic equilibrium requiring the animal to make some responses to maintain its psycho-physiological integrity [18]. The life of a farm animal is constantly challenged by an array of factors that may evoke stress responses. Overcrowding, extreme temperatures, social disruption, unfamiliar sounds, unfamiliar or uncaring handlers, feed and water restriction, injection with antigens, disease are common environmental factors that may disrupt homeostasis. Biological reactions to stress comprise changes in behaviour, neuroendocrine system, autonomic nervous system and immune system [19]. In many stressful events, the first line of defence is behavioural response, which is biologically economic. When the biological system fails to cope with the stressor(s) and behavioural activity is suppressed, an animal depends on the integrative capacities of nervous and endocrine systems. Regardless of whether the stimulus is threatening or not, two distinct pathways involving interlocking physiological reactions will be evoked. The first pathway comprises the sympathetic adreno-medullary (SA) system that is responsible for the increase in the synthesis and release of cathecolamines (adrenaline and noradrenaline). The SA system reaction is manifested by immediate increases in blood pressure, muscle tone, nerve sensibility, respiration rate and blood sugar. Although the system may have dramatic physiological consequences, it is short term. Activation of the hypothalamic-pituitary-adrenal axis is a longer adjustment to environmental fluctuations. Sensory inputs cause the release of corticotrophin-releasing hormone form the hypothalamus. The neurohormone stimulates the anterior pituitary gland to release adrenocorticotrophin hormone that elicits the adrenal cortex to release glucocorticoids (cortisol and corticosterone). Glucocorticoids are known to modulate immune response, shift metabolism, influence growth, and alter behaviour [20]. Changes in the circulating levels of cortisol or corticosterone are routinely used to measure an animal’s response to stress.

Stress and fear are not synonymous but the latter may contribute to overall stress, particularly if the frightening stimulation is intense, prolonged or inescapable [21,22]. According to Jones [8], fear is an emotional (psychophysiological) response to perceived danger. Gray [23] defined fear as a form of emotional reaction to a stimulus that the animal works to terminate, escape from, or avoid. High levels of fear not only represent a state of suffering but they are also a powerful and potentially damaging stressor. Two of the commonest and potentially frightening events encountered by farm animals are sudden changes in their social or physical environment and their exposure to people [8]. Animals probably perceive a new unfamiliar environment with a degree of uncertainty that acts as a psychological stimulus. In pigs, short-term exposure to a novel environment induced both behavioural and emotional reactions such as increased locomotive activity and escape attempts, vocalization, and as well as hormonal responses [24]. Novel environment is a potent fear- and stress-elicitor in all animals. Zulkifli et al. [25], and Zulkifli and Siti Nor Azah [26] noted elevation of heterophil / lymphocyte ratios (HLR) 24 h following transfer of chicks to new home cages. Translocation of chicks from the hatcher to brooding cages or pens may result in behavioural inhibition and panic [8].

The predominant response of the domestic fowl to humans is thought to be one of fear [27]. Naive chickens may perceive contact with humans as an alarming predatory encounter. It is common for farm animals to display fear-related behaviour in the presence of humans such as withdrawal from or avoidance of humans, immobility such as freezing or crouching [9,21]. Fear of humans in farm animals can be measured by home cage avoidance test, box plus experimenter and approaching human test [8]. The approaching human test is useful for commercial poultry flocks raised in floor pens. An experimenter can film the withdrawal responses of chickens as he walks slowly through a chicken house. Orientation away and withdrawal from the approaching human may be equated with fear levels. It is well documented that procedures with high human involvements, such as catching, loading and unloading can evoke both stress and fear reactions which may compromise chickens’ welfare [28-30]. Hemsworth and Gonyou [5] indicated that pigs exhibited marked avoidance of humans following imposition of daily negative interactions as little as 15 to 30 s.

There is considerable report to suggest that regular positive human contact is a powerful and reliable method to dampen stress and fear reactions in pigs [31], dairy cows [32], goats [33], and poultry [26,34,35]. Al-Aqil et al. [35] subjected broiler chicks to a pleasant physical contact daily for 30 s from 1 to 28 d of age. The authors found that those chickens had lower HLR, plasma levels of corticosterone (CORT), and shorter tonic immobility (TI) duration than their neglected counterparts following road transportation. Jones [8] suggested that the benefit of regular handling was specifically reducing birds’ fear of humans rather than through any effect on their underlying fearfulness. However, during transit chickens may be exposed to an array of stressful and fearful stimuli including thermal extremes, acceleration, vibration, motion, impacts, feed and water deprivation, social disruption and noise [36]. Similarly, Lyons [37] reported that early human contact not only influenced behavioural responses to humans but also to novel stimuli. Hence, the findings of Lyons [37], and Al-Aqil et al. [35] showed that regular pleasant human contact may attenuate nonspecific underlying fearfulness in animals.

There is evidence that poultry are also sensitive to visual contact with humans [26,38,39]. Zulkifli et al. [34] reported that visual contact procedure involving the experimenter standing in the centre of a pen (with no attempted physical contact with birds) for 10 min twice daily from 0 to 3 wk reduced fear and stress reactions to handling and crating. Jones [38] demonstrated that visual contacts without tactile interaction was more effective in reducing fear of humans than picking up and stroking the birds. Visual contact is obviously more feasible and practical than physical contact in commercial poultry flocks. There is, however, limited documented work on visual contact with humans, on stress and fear responses in non-avian species.

One of the earliest studies on the effect of early age stimuli on physiological stress response was by Levine [40] who reported that infantile stimulation through handling elicited long lasting alterations of the adrenocortical function in rodents. When adults, these rats had lower CORT both basally and during recovery, after withdrawal of stressors than those that were not handled, Gross [41] suggested that stimulation which occur early in life while many systems of the animals are still developing may have long lasting impact and could possibly modify expression of their genetic potential. Studies in pigs demonstrated that early handling during the first eight wk of life increased the approach behaviour of pigs to an experimenter in a standard test from 10 to 24 wk of age [42]. Zulkifli et al. [34] compared the effect of regular visual contact from 0 to 3 wk, 0 to 6 wk and 3 to 6 wk in chickens subjected to crating at 42 d of age. Chickens subjected to visual contact from 3 to 6 wk showed longer TI durations and higher HLR in response to crating than those interacted at other ages. Based on these studies, it appears that early age human contact may have long-term effects. On the other hand, Jones and Waddington [43] reported that fear of humans in 20-day-old chicks was equally reduced irrespective they were handled from 0 to 9, 10 to 18, or 0 to 18 d of age.

It is not clear whether the quality of human contact experienced by animals at an early age can be modified by subsequent pleasant or unpleasant interaction with humans. This is critical under commercial setting because there will be variation both between and within stockpersons in their behaviour toward farm animals. Al-Aqil et al. [35] subjected chicks to either a combination of pleasant-unpleasant or unpleasant-pleasant physical contacts from 1 to 14 d and 15 to 28 d of age, respectively. Based on HLR and CORT reactions to road transportation, the authors concluded that the benefits of early age positive human contact can be modified by subsequent unpleasant experience with humans. The authors also indicated that chickens which had experienced pleasant human contact early in life may perceive the presence of humans as a signal for continuous positive interaction. Hence, subsequent exposure to unpleasant human contact may result in disappointment with consequent elicitation of the physiological stress response.

### Effect of human-animal interactions on animal productivity

There is substantial evidence of a negative relationship between underlying fearfulness and productivity in farm animals [4,5]. Because positive interaction can reduce fear of humans, such practice may enhance productivity of farm animals. Gross and Siegel [44] postulated that positive human contact may reduce the resources otherwise required by animals to respond to their human associates and that resources can be utilised for productivity. In poultry, some authors [44-46] reported a significant improvement in weight gain and feed efficiency in positively handled chickens. The enhanced disease resistance and immune response in those studies could be associated with the stress modulating effect of human contact. However, others demonstrated that positive tactile interaction either had negligible [47] or negative effect [48] on growth performance. Nature of the physical contact, breed and age differences may have accounted for the discrepancies. Zulkifli et al. [34] reported that regular visual contact, irrespective of age, had no effect on weight gain, feed intake, FCR and mortality rates of broiler chickens. Zulkifli and Siti Nor Azah [26] compared the effects of physical and visual contacts and showed only the former was beneficial in enhancing growth performance. Physical contact which involved picking up and stroking the chickens appeared to be more “interactive” than visual contact in broiler chickens. In laying hens, however, Barnett et al. [39] showed that regular visual contact which reduced the subsequent avoidance behaviour of laying hens improved egg production.

According to Hemsworth and Coleman [26], fear of humans may be considered as one of the major factors for depressed growth and reproductive performance in commercial pigs. Hemsworth et al. [31] subjected gilts to either pleasant or unpleasant human contact three times per week for 2 min in duration from 11 to 22 wk of age. The authors noted gilts in the pleasant handling treatment had significantly better weight gain but not feed efficiency than those in the unpleasant handling treatment. Unpleasant physical contact with humans reduced testicle size and delayed co-ordinated mating response in boars when compared to those subjected to positive handling by humans. In the similar study, gilts in the unpleasant treatment showed a lower pregnancy rate than those in the pleasant treatment. Work by Paterson and Pearce [49], and Pearce et al. [50], however, suggested that the growth response of pigs housed in groups was not affected by regular aversive handling by humans. There is a possibility that pigs raised in large groups may receive psychological protection from members of the group.

There have been relatively few human contact and productivity studies in other farm animals. Rushen et al. [51] showed that pleasant human contact had negligible effect on milk yield but reduced some behavioural signs of agitation in dairy cattle that were stressed due to milking in an unfamiliar environment. The authors concluded that human contact is not sufficient to reduce neuroendocrine reaction to novelty / isolation stress.

### Effect of human-animal interactions on animal health

The immune system, once considered an autonomous system, is integrated with other physiological systems and is sensitive to regulation of the brain [52]. Because human contact may result alterations in brain physiology and morphology [53] it is possible that immune response and disease resistance will be affected. It is well established that farm animals encountering challenging conditions often show some degree of immunosupression [54-57]. Chronic activation of the hypothalamic-pituitary-adrenal axis and the sympathetic-adrenal-medullary axis results chronic production of corticosteroids and cathecolamines, respectively. Lymphocytes, monocytes or macrophages and granulocytes, exhibit receptors for corticosteroids and cathecolamines, which can alter cellular trafficking, proliferation, cytokine secretion, antibody production and cytolytic activity [58].

Because fear is a potent stressor, reducing fear of humans in farm animals through positive human contact may enhance the health of animals. Other than poultry there is a lack of information on the effect of human contact on the immune response and disease resistance in farm animals. Gross and Siegel [44,59,60] reported that chickens habituated to humans through pleasant contact were more resistant to *Escherichia coli* and *Staphylococcus aureus* infections and had greater antibody production against erythrocyte antigens than those that were ignored. Zulkifli et al. [34] indicated that regular visual contact from 0 to 3, and 0 to 6 wk of age may improve antibody production against Newcastle disease vaccine. Similarly, Barnett et al. [39] showed that hens subjected to regular pleasant human contact had improved cell-mediated immune response to a mitogen when compared to the chickens that received negative human contact. The benefit of human contact improving disease resistance and immune response could be associated with its effect on modulating physiological stress response.

### How do positive human-animal interactions improve animal productivity and welfare?

There is the question of how positive human-animal interaction can improve productivity and modify physiological stress response of farm animals. At any particular time, resources available to an individual are finite. Hence, competition for resources between body functions such as growth, reproduction and health will always occur [57]. The resources required to respond to prolonged and severe stress may be significant. Gross and Siegel [9] suggested that habituation to humans reduces the resources otherwise needed by the bird to respond to subsequent human contact, and these resources could be used either for coping ‘with other environmental stressors of for productivity.

Jones [8] suggested that regular human contact exerts its effect by specifically reducing chickens’ fear of humans. For example regular handling failed to influence chicks’ reactions to unfamiliar places and objects [61,62]. On the contrary, Lyons [37] reported that early human contact not only influenced behavioural reaction of goats to human exposure but also a range of novel stimuli. Al-Aqil et al. [35] showed that regular pleasant human contact reduced stress and fear reactions to road transportation in broiler chickens. Although road transportation involved handling by humans, it is a multifactorial process which include feed and water deprivation, noise, vibration, thermal extremes, social disruption, crowding and restriction of movements [36]. Fluck [63] suggested that handling of chicks decreased forebrain aminobutyric acid (GABA) receptors and in vitro GABA release from brain tissues. Hence, it appears that reduced chickens’ fear to humans is not the only possible explanation for the benefit of a positive human-animal interaction on farm animals.

When living organisms are exposed to thermal stresses, the synthesis of most proteins is retarded but a group of highly conserved proteins known as heat shock proteins (hsp) are rapidly synthesized [64]. In a heat shocked cell, hsp may bind to heat sensitive proteins and protect them from degradation, or may prevent damaged proteins from immediately precipitating and permanently affecting cell viability. It has been documented that stressors other than thermal stressors, for example feed restriction, confinement in crates, transportation and social isolation [65-68] may also elicit hsp 70 response in poultry. The induction of hsp is often associated with increased tolerance to environmental stressors and disease resistance [65,69]. Al-Aqil et al. [35] subjected broiler chickens to either pleasant or unpleasant negative human handling from 1 to 28 d of age. Following 3 h of road transportation, the chickens had lower heterophil / lymphocyte ratios, shorter TI duration and greater hsp 70 expression than those that were ignored or handled unpleasantly. Thus, it can be concluded that pleasant human contact may alter ability to tolerate road transportation stress through enhanced hsp 70 expression.

### The impact of stockmanship on animal productivity and welfare

The factors commonly emphasised to improve farm animals’ productivity are genetics, housing, nutrition, and health. There is, however, less emphasis on the quality of stockmanship. The way a stockperson carry out his or her routine animal care tasks may contribute to the overall relationship that animals have with humans and determine the relationship is positive, negative or neutral. Review of research in commercial pig and dairy farms showed a significant sequential relationships between stockpeoples’ attitudes and behaviour towards animals and the fear of humans and productivity [70,71]. However, there is limited work in the poultry industry. Cransberg et al [72] investigated the relationship between stockperson attitude and behaviour, bird behaviour and productivity in 24 commercial broiler chicken farms. Unlike findings in pigs and dairy cattle, the authors failed to find a relationship between stockpeoples’ attitude and behaviour. Although sequential relationships between human behaviour, bird behaviour and production were noted by the authors the magnitude of these relationships were not as substantial as found in pig and dairy industries. The lack of physical contact between stockperson and chickens and the large number of chickens managed by the stockpeople explanations for the results [72]. On the contrary, close physical contact between stockpeople and animals is common in pig and dairy farms.

The attitude of a stockperson holds about animals will strongly influence their behaviour towards animals [4]. The attitude and consequent behaviour of stockpeople affect the animals’ fear of human which eventually influence animals’ productivity and well-being. Other than attitude and behaviour, technical skills, knowledge, job motivation, commitment and job satisfaction are prerequisites for high job performance. Hence, proper selection and formal training of stockpeople are critical. Today, many countries, including Malaysia, are facing labour shortage in the agriculture sector. This may limit the capacity to select high potential stockpersons. Another possible limiting factor is the inadequate educational background of the stockpeople which may restrict their ability to be trained formally [4]. Thus, the content of the training programme has to be easily comprehensible by the target group. It is also critical that the animal industry recognizes and appreciates the stockpeoples’ role in determining animal performance and welfare. Better financial rewards and clear career pathway for stockpeople would contribute to better motivation and job performance.

## Conclusion

The preceding discussion clearly highlights the opportunity to improve productivity and welfare of farm animals through positive human-animal interactions. Most of the previous findings on human-animal interaction were based on laboratory studies. More on-farm studies, particularly in poultry, are required before appropriate operational strategies can be formulated for overall ease and feasibility of implementation in commercial settings. Factors such as genetic background, housing system, prior experience and individual variation may determine how an animal respond to human contact. The precise physiological mechanisms underpinning the effect of human-animal interactions on productivity and welfare of farm animals are unclear, although changes in ability to express heat shock proteins may be considered a possible route of action. The quality of stockmanship may markedly influence the productivity and welfare of farm animals. In intensive animal productions systems the tasks of managing and monitoring intensively-raised animals have been increasingly replaced by the use of modern technologies such as automation and surveillance cameras. Such technologies are labour-saving but opportunities for animals to interact with humans will be limited and thus, may exacerbate their natural fear of humans. A more regular visual contact with animals is necessary to dampen underlying fearfulness and physiological stress in farm animal. Hence, the attitude and behaviour of the stockpeople towards farm animals have to be emphasised.

## Competing interests

The author declares that he has no competing interests.
